# Outbreak of lassa fever in Nigeria: measures for prevention and control

**DOI:** 10.11604/pamj.2016.23.210.8923

**Published:** 2016-04-20

**Authors:** Kehinde Charles Mofolorunsho

**Affiliations:** 1 Department of Microbiology, Faculty of Natural Sciences, Kogi State University Anyigba, Nigeria

**Keywords:** Lassa virus, haemorrhagic fever, mastomys natalensis, Nigeria

## To the editors of the Pan African Medical Journal

Lassa fever, an acute viral haemorrhagic fever, extremely virulent and often infectious, occurs very frequently in different parts of Nigeria [1–4] and affects approximately 100,000-500,000 persons per year in West Africa [5, 6]. The illness was discovered in Lassa, Borno State where it was first reported. It is caused by the Lassa fever virus, a single stranded RNA virus belonging to the *arenaviridae* family [7, 8].

The incubation period for Lassa fever varies from 6 – 21 days. It is symptomatic and usually characterized by fever, myalgia, nausea, vomiting, sore throat, abdominal and chest pains. Illness may progress to more serious symptoms including haemorrhaging, neurological problems, hearing loss, tremors and encephalitis [8–10].

Lassa virus is zoonotic and infected rodents in the *mastomysnatalensis* species complex are reservoirs capable of excreting the virus through urine, saliva, excreta and other body fluids to man [7, 11]. Secondary human – to- human spread within a community may occur through inhalation or ingestion. Nosocomial transmission is also not uncommon [10, 12].

Lassa fever is endemic in parts of West Africa including Sierra Leone, Liberia and Nigeria [10]. Outbreaks of the disease have been reported in various parts of Nigeria and the most recent of them is the on-going 2016 outbreak. There have been several Lassa fever outbreaks since it was first reported in 1969 with the worst outbreak recorded in 2012 where 623 cases including 70 deaths were reported from 19 out of the 36 states [8, 9, 13, 14]. In the current outbreak, cases are so far being reported in Seventeen states with 212 suspected cases and 63 deaths. Case fatality rate has been put at 37.9% [15].

Since Lassa fever presents with no specific symptoms, clinical diagnosis is often difficult especially at the early onset of disease. Accurate diagnosis therefore can be assisted with differential laboratory testing, clinical manifestations, epidemiological findings since definitive diagnosis requires investigations available only in highly specialized laboratories [13, 16].

Early treatment of Lassa fever is very important for survival and requires specialized treatment using the guanosine analogue ribavirin. Care must also be taken to avoid spread of the disease in hospital settings [14, 17].

Due to the absence of vaccine against the virus and the impractical control of the rodent host (*mastomys species*) population, control measures are limited to keeping rodents out of homes and food supplies and also maintaining proper personal hygiene. Using these rodents as food source should be discouraged [5, 18]. Enlightenment and awareness of the public on risk factors associated with spread of disease is important for prevention. Protective measures should be put in place to reduce human infection. Infected persons should be isolated and their body fluids and excrements properly disposed. Healthcare workers should take proper precautions in order to curtail nosocomial spread of disease through the use of Personal Protective Equipment (PPE).

## Conclusion

In conclusion, the current outbreak of Lassa fever in Nigeria can be controlled effectively by adopting proper standard precautions in hospitals as well as communities. Educating the public on the mode of transmission of this virus and the need for proper hygiene and environmental sanitation should be emphasized.
